# Previously reported *CCDC26* risk variant and novel germline variants in *GALNT13*, *AR*, and *MYO10* associated with familial glioma in Finland

**DOI:** 10.1038/s41598-024-62296-5

**Published:** 2024-05-21

**Authors:** Riikka Nurminen, Ebrahim Afyounian, Niina Paunu, Riku Katainen, Mari Isomäki, Anssi Nurminen, Mauro Scaravilli, Jenni Tolppanen, Vidal Fey, Anni Kivinen, Pauli Helén, Niko Välimäki, Juha Kesseli, Lauri A. Aaltonen, Hannu Haapasalo, Matti Nykter, Kirsi J. Rautajoki

**Affiliations:** 1 Prostate Cancer Research Center, Faculty of Medicine and Health Technology, Tampere University, Arvo Ylpön katu 34, 33520 Tampere, Finland; 2 Tays Cancer Center, Tampere University Hospital, Tampere, Finland; 3 Department of Oncology, Tampere University Hospital, Tampere, Finland; 4 Applied Tumor Genomics Research Program, Department of Medical and Clinical Genetics, Faculty of Medicine, University of Helsinki, Helsinki, Finland; 5 Fimlab Laboratories ltd., Tampere University Hospital, Tampere, Finland; 6 Foundation for the Finnish Cancer Institute, Tukholmankatu 8, Helsinki, Finland; 7 Tampere Institute for Advanced Study, Tampere University, Tampere, Finland

**Keywords:** Cancer genomics, Cancer genetics

## Abstract

Predisposing factors underlying familial aggregation of non-syndromic gliomas are still to be uncovered. Whole-exome sequencing was performed in four Finnish families with brain tumors to identify rare predisposing variants. A total of 417 detected exome variants and 102 previously reported glioma-related variants were further genotyped in 19 Finnish families with brain tumors using targeted sequencing. Rare damaging variants in *GALNT13*, *MYO10* and *AR* were identified. Two families carried either c.553C>T (R185C) or c.1214T>A (L405Q) on *GALNT13*. Variant c.553C>T is located on the substrate-binding site of *GALNT13*. *AR* c.2180G>T (R727L), which is located on a ligand-binding domain of AR, was detected in two families, one of which also carried a *GALNT13* variant. *MYO10* c.4448A>G (N1483S) was detected in two families and c.1511C>T (A504V) variant was detected in one family. Both variants are located on functional domains related to *MYO10* activity in filopodia formation. In addition, affected cases in six families carried a known glioma risk variant rs55705857 in *CCDC26* and low-risk glioma variants. These novel findings indicate polygenic inheritance of familial glioma in Finland and increase our understanding of the genetic contribution to familial glioma susceptibility.

## Introduction

Gliomas account for approximately 25% of all primary brain and other central nervous system (CNS) tumors (collectively called brain tumors from this point onwards) and approximately 75% of malignant primary brain tumors^1^. Although gliomas are rare, higher grade gliomas are extremely fatal, with a 5-year survival rate of 5.6%^1^. Only a few well-established etiological factors have been identified, including increased risk after ionizing radiation^2^ and decreased risk associated with allergic condition factors^3^.

Familial aggregation of glioma cases^4^, the twofold higher glioma risk in relatives of glioma patients^5^ and the association of^4^ rare hereditary cancer syndromes with glioma cases^6^ suggest a contribution of hereditary components to disease development. We have identified in the Finnish registry-based study that the risk of diffuse glioma, and especially the risk of early-onset diffuse glioma, is elevated in the 1st degree relatives of early-onset diffuse glioma probands, which further supports the findings of inherited genetic factors underlying the increased risk^7^. However, the low incidence^1^, histologic heterogeneity and suggested polygenic nature of this disease^4,8,9^ have hampered the discovery of predisposing factors. The candidate gene approach has not resulted in replicated findings^10^, and common low-risk single-nucleotide polymorphisms (SNPs) identified through genome-wide association studies (GWASs) have explained only 27–37% of the inherited disease risk, depending on histologic subtype^8^. Similarly, the associated rare cancer syndromes explain only a very small part of the glioma occurrence^4^.

Previously, the hereditary component of familial glioma was studied in 24 Finnish families with brain tumors^11^. None of the families fulfilled the classical tumor syndrome criteria^11^ or carried variants in the Li-Fraumeni syndrome predisposition gene *TP53*^12^. These facts suggest that still unknown predisposing factors underlie the clustering of gliomas in these families. To identify variants that contribute to increased glioma risk in these Finnish families with brain tumors, we performed family-based whole-exome sequencing (WES) of blood DNA to identify rare protein-altering variants and analyzed the detected variants together with previously reported variants in a larger Finnish family cohort with targeted DNA sequencing.

## Methods

### Study cohort

The study included 19 of the 24 originally described Finnish families with brain tumors^11^. Briefly, the glioma families were identified through questionnaires sent to 369 consecutive glioma patients operated at Tampere University Hospital during 1983–1994. Twenty-four families with 55 verified glioma patients were identified. The 19 families had 42 patients with gliomas confirmed from medical records. We had access to hospital records of 25 glioma patients to survey remarks on unusual family history and certain key manifestations encountered in other tumor syndromes. In these families, there were no optic gliomas or other clinical signs of neurofibromatosis 1. Two patients had spinal ependymomas (a feature of neurofibromatosis 2), but no other signs such as (vestibular) schwannomas, multiple meningiomas, multiple ependymomas, glial hamartias, cerebral calcifications or ocular lesions were found. One subependymal giant cell astrocytoma (a sign of tuberous sclerosis) was also diagnosed, but no remarks of cortical hamartomas, cutaneous angiofibromas or intellectual disability were found^12^. Samples were collected at Tampere University Hospital, Tampere, Finland, and the collection process has been described earlier^11^. Informative blood samples were available from 19 families. All individuals from whom the samples were derived were of Finnish origin. Table 1 presents the characteristics of the families and the brain tumor-affected family members, which were included in the WES and targeted sequencing analyses. Each of the families had two to five brain tumor cases (Table 1), and 11 of the families (58%) included one (8/11 families) or two (3/11 families) childhood or adolescence brain tumor cases (onset at age < 20 years old). For the WES analysis, we selected families in which there was a blood DNA sample available from at least two glioma patients as well as data from either or both of parents of the patient(s) to estimate the segregation of the variants. When available, we also included siblings of glioma patients into the analysis, resulting in a total of 13 blood samples from four families that were analyzed with WES. Power analysis revealed a minimum detection power of 53% for identifying a variant that is present in at least 20% of the families with this approach (see Supplementary Information for details).

**Table 1:** Familial glioma study cohort.

	WES and targeted sequencing (n)			Targeted sequencing (n)		
	Families	Affected cases	Unaffected relatives	Families	Affected cases	Unaffected relatives
Total	4	8	5/13^b^	15^c^	15	44
Cases (n) with all types of CNS tumors^a^						
2	1			11		
3	2			2		
4	0			1		
5	1			1		
Cases (n) with glioma tumors		8			15	
1				4		
2	4			8		
3				1		
4				2		
Grade I and II gliomas		8			13	
Grade IV gliomas		0			2	
Age of diagnosis (y)						
< 20		4			6	
20–40		4			7	
40–60		0			2	

*CNS* central nervous system, *WES* whole-exome sequencing.

^a^Gliomas are included in total number of CNS tumors; ^b^WES/targeted sequencing; ^c^includes two families with no samples available from affected individuals. Only unaffected family members (n = 6) were included in the study from these families.

After the initial screening, targeted DNA sequencing was used to genotype the variants/selected genomic regions from blood samples originating from 23 affected glioma cases and 57 unaffected relatives. At this phase, we included an additional 15 affected glioma cases and 44 unaffected relatives belonging to 15 new families. A sample was available for sequencing from one glioma-affected individual from the majority of the newly added families (11 of 15). Samples from two glioma patients were available from two families outside the WES cohort. No samples from affected individuals were available from two of the families; thus, samples from unaffected relatives were included in the analysis instead. In addition, 11 samples from the WES cohort and 10 additional samples from unaffected individuals belonging to the exome sequenced families were analyzed with targeted sequencing. For further inspection of the identified genomic alterations and candidate genes, formalin-fixed paraffin-embedded (FFPE) glioma tumor samples and matched blood samples of eight glioma patients belonging to eight different families were analyzed using whole-genome sequencing (WGS) (Supplementary Table 1). In addition, only blood samples from three glioma patients were included in WGS. All samples originated from individuals who were originally analyzed by WES and/or targeted DNA sequencing.

The frequencies of most interesting genomic variants detected in the Finnish familial glioma cohort were analyzed in the germline WGS data of glioma patients in the Pan-Cancer Analysis of Whole Genomes (PCAWG) project^13^. The data of 149 glioma patients from the Brain Glioblastoma Multiforme—TCGA, US (GBM-US), Brain Lower Grade Glioma—TCGA, US (LGG-US) and Pediatric Brain Cancer—DE (PBCA-DE) projects with the DKFZ/EMBL variant calling pipeline were downloaded from the International Cancer Genome Consortium (ICGC) data portal^13,14^. Somatic alterations in *GALNT13*, *AR*, and *MYO10* that have been reported in The Cancer Genome Atlas (TCGA) diffuse glioma^15^ or Glioma Longitudinal AnalySiS (GLASS) consortium^16^ dataset were inspected from cBioPortal^17,18^.

The study was conducted in accordance with the Declaration of Helsinki and official regulations. Full written informed consent concerning the sample and patient information was obtained from the participants. The study was conducted with appropriate research permissions from the Ethics Committee of the Tampere University Hospital, Finland (R18069, 12.6.2018–31.12.2028 and R07042, 30.3.2007–31.12.2024); Ministry of Social Affairs and Health, Finland (STM/2581/2005, 2.2.2007); Population Register Centre, Finland (VRK/5577/2018-2); Valvira, Finland (V/78697/2017, 17.11.2017); and Finnish Social and Health Data Permit Authority Findata, Finland (THL/2454/14.02.00/2021, 2.6.2022).

### Sample preparation and DNA sequencing

Genomic DNA was extracted from the blood with the Puregene DNA Isolation Kit according to the manufacturer's protocol (Gentra Systems, Inc., Minneapolis, MN, USA). Genomic DNA was extracted from FFPE tumor samples using a GeneRead DNA FFPE Kit according to the manufacturer’s instructions (Qiagen, Valencia, CA, USA). FFPE samples contained more than 50% of tumor cells based on the pathologist’s evaluation. WES, targeted DNA sequencing, WGS and TaqMan SNP Genotyping Assay are described in the Supplementary Information.

### Sequencing data analyses

**Figure 1:**
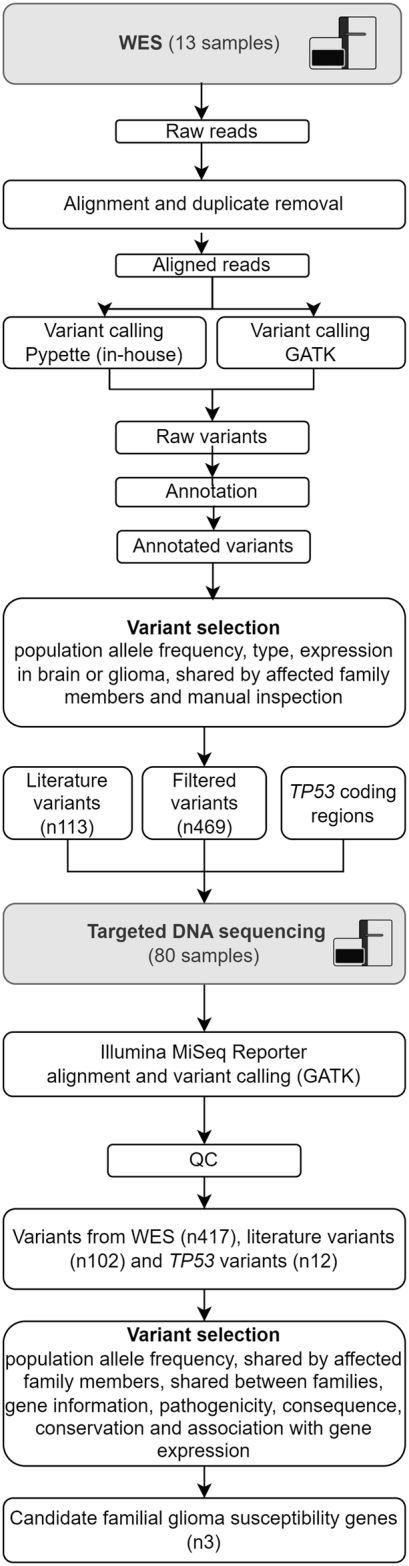
Whole-exome sequencing (WES) and targeted sequencing data analysis workflow. WES was conducted on 13 individuals of four Finnish glioma families. Germline variants were called using an in-house developed and GATK pipeline and filtered based on population allele frequency and functional annotation. Variants shared by affected members in at least one family were sequenced in additional 15 glioma families together with variants described in glioma articles (i.e., literature variants). Targeted sequencing variants were called using the GATK pipeline. Quality control procedure was done on genotype data and the variants were evaluated in the glioma families. A total of three genes with rare, damaging variants were identified in the Finnish glioma families. Abbreviations: *n* number of variants, *QC* quality control.

The overall workflow of the WES and targeted sequencing data analyses are presented in Fig. 1. Briefly, 13 individuals in the cohort (from 4 different families) underwent WES and the data was analyzed to detect germline variants using two different pipelines. After variant filtration, 469 variants, together with 113 variants extracted from literature, and *TP53* coding regions underwent targeted sequencing in 80 individuals. Pre-processing and analysis of the targeted data resulted in a list of three candidate familial glioma genes. As a complementary approach, the called WES variants were reanalyzed to discover whether there were glioma-segregating, predicted damaging and very rare variants in genes that were the most relevant in glioma and/or the brain based on the published literature. WGS data were analyzed to detect germline variants and somatic mutations. Detailed description of WES, targeted sequencing, complementary WES and WGS data analyses are presented in Supplementary Information.

## Results

To detect candidate predisposing variants, we performed WES on blood-derived DNA from 13 participants (including both affected n = 8 and unaffected n = 5 individuals) from four unrelated families. We used an in-house pipeline based on sequencing read pileup, as well as the genome analysis toolkit (GATK) pipeline to call 8,263,156 and 210,026 raw germline variants, respectively, including SNPs and short insertions and deletions (indels). No significant familial glioma-associated germline copy number alterations were detected based on the WES data. After careful filtering of WES SNP and indel data (Fig. 1), we identified 469 variants, which were genotyped together with coding regions of the *TP53* gene in 15 additional families using targeted sequencing. Based on the relevant literature, we included 113 additional variants in targeted sequencing. After quality control, 417 variants from WES, 102 literature variants and 12 *TP53* variants were included in the subsequent steps.

Approximately 70% (n = 293) of the variants analyzed with targeted sequencing were detected in only one exome-sequenced family in at least one affected individual. Approximately 18% (n = 76) of the WES-derived variants were detected in at least one affected individual in one additional family and 9% (n = 36) in two or more additional families in addition to an exome-sequenced family or families. Most variants (n = 329, 79%) were annotated as missense alterations (Supplementary Information, Fig. 1). We prioritized variants that were rare in the Finnish population (< 0.01 Finnish population allele frequency), were shared between affected family members, occurred in affected individuals in at least two families with brain tumors, were inherited from the brain tumor side, were predicted to have an effect on gene function, and were located on a gene that is expressed in brain and/or glioma tumors or that has other reported relevance in glioma based on the literature. All criteria were required for a variant to be prioritized. With these criteria, we identified rare, predicted pathogenic variants in the genes *polypeptide N-acetylgalactosaminyltransferase 13 (GALNT13)*, *androgen receptor (AR)*, *and myosin X (MYO10*) (Table 2). Two different, rare, damaging variants that fulfilled the prioritization criteria were found for *GALNT13*. Different variants fulfilling the prioritization criteria were not detected in any additional genes analyzed in the study.

**Table 2:** Rare variants of *GALNT13*, *AR*, and *MYO10* in Finnish glioma families.

Variant position (hg19)	Gene	HGVS coding ID (protein ID)	Family (n) (ID)	Affected cases (n)	Unaffected cases (n)	MAF^a^	Predicted to be damaging by an algorithm (n/total tested)^b^	CADD phred	Conservation score^c^
chr2:155099285	*GALNT13*	c.553C>T (p. R185C)	1 (A)	2	1	8.0e−5/7.7e−6/1.8e−5	1,2,3,4,5,6,7,8 (8/8)	31.0	5.6/1.1/15.1
chr2:155252560	*GALNT13*	c.1214T>A (p.L405Q)	1 (C)	2	1	4.6e−5/0/4.0e−6	1,2,3,4,5,6,7,8 (8/8)	27.7	5.0/7.9/13.9
chr5:16680150	*MYO10*	c.4448A>G (p.N1483S)	2 (B, J)	3	3	9.3e−5/0/8.0e−6	2,4,5,6,7,8 (6/7)	23.0	4.2/1.7/12.8
chr5:16762730	*MYO10*	c.1511C>T (p.A504V)	2 (F, O)	1	2	1.6e−3/8.3e−5/2.0e−4	1,2,4,5,6,7,8 (7/7)	29.3	5.6/9.6/19.7
chrX:66937326	*AR*	c.2180G>T (p.R727L)	2 (A, Q)	3	3	8.4e−3/3.1e−4/9.6e−4	1,2,3,4,6,7,8 (7/7)	29.7	4.9/9.9/14.5

No ClinVar annotations available with review status ≥ 2/4 stars (i.e., criteria provided, multiple submitters, no conflicts) for the variants.

*CADD* Combined Annotation Dependent Depletion, *HGVS* The Human Genome Variation Society, *MAF* minor allele frequency.

^a^Finnish population/Non-Finnish European population/global population allele frequency derived from gnomAD.

^b^1 = SIFT, 2 = PolyPhen, 3 = LRT, 4 = MutationTaster, 5 = MutationAssessor, 6 = FATHMM_MKL, 7 = MetaSVM, 8 = MetaLR. No LRT predictions were available for *MYO10* c.4448A>G and c.1511C>T and no MutationAssessor prediction for *AR* c.2180G>T.

^c^GERP++_RS/phyloP100way_vertebrate/SiPhy_29way_logOdds.

### Two distinct rare *GALNT13* variants detected in two different families

**Figure 2:**
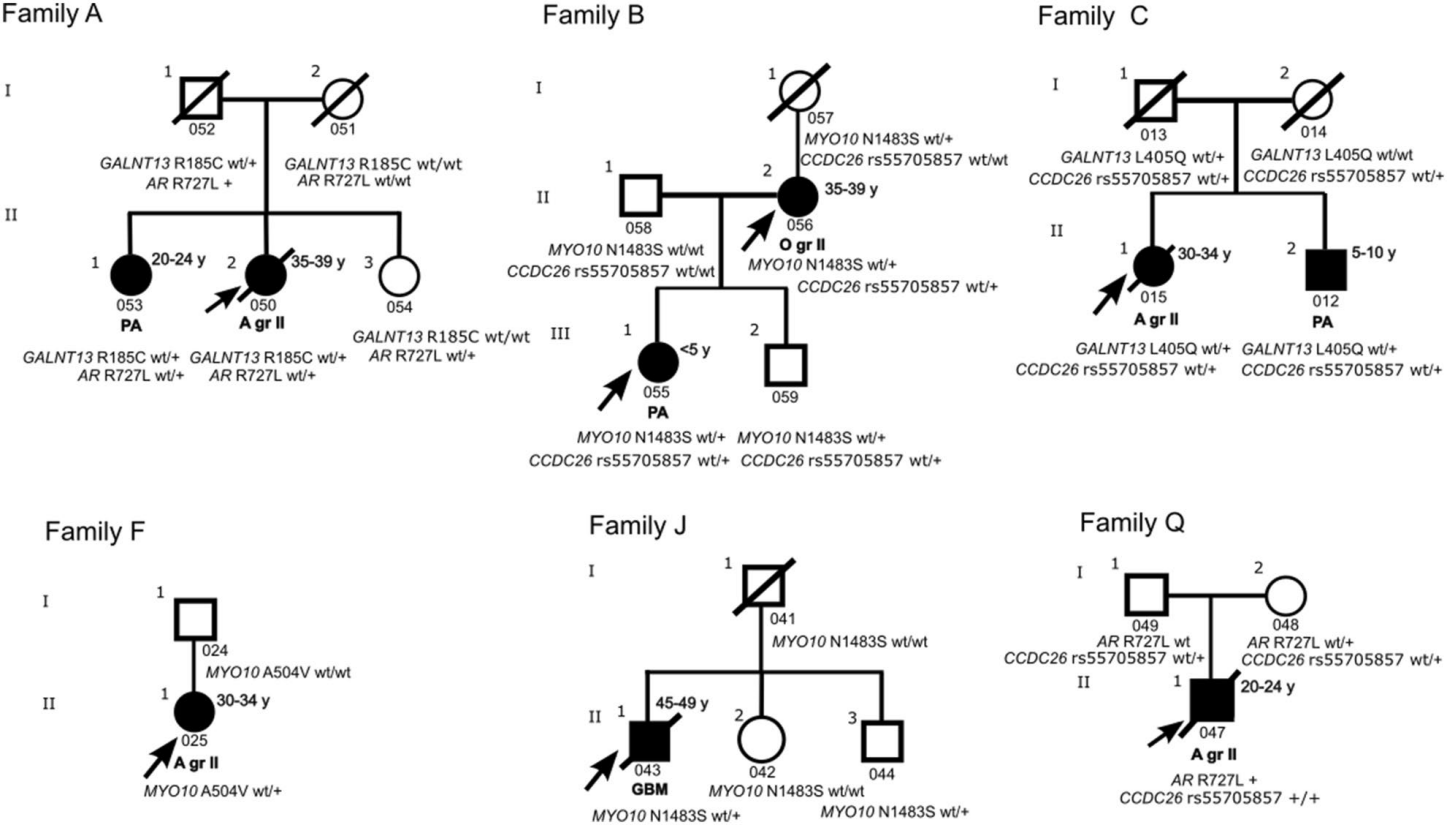
Segregation of *CCDC26*, *GALNT13*, *AR*, and *MYO10* variants in the Finnish families with brain tumors. Two families carried either R185C (c.553C>T) or L405Q (c.1214T>A) on *GALNT13*. *MYO10* was mutated in affected individuals in three families. Two families carried *MYO10* N1483S (c.4448A>G), and *MYO10* A504V (1511C>T) was detected in one family. Two families carried the *AR* variant R727L (c.2180G>T). One family carried both the *GALNT13* R185C (c.553C>T) and *AR* R727L (c.2180G>T) variant. *CCDC26* variant (rs55705857) was detected in affected individuals in three families, which also carried either *GALNT13*, *MYO10* or *AR* variant. A variant allele is denoted by a plus sign (+), and wt abbreviation signifies a wild type allele. As *AR* is located on chromosome X, only + sign or wt abbreviation is used for the male individuals. Circles denote female participants, and squares signify male participants. Symbols filled with color black indicate a patient with a brain tumor. Age range at diagnosis in years (y) is presented next to the symbol. Deceased family members are denoted with a diagonal line and index patients with an arrow. Sample ID is presented below the symbol. Only individuals who gave written informed consent are presented in the pedigrees. Abbreviations: *A* astrocytoma, *GBM* glioblastoma, *gr* grade, *O* oligodendroglioma, *PA*  pilocytic astrocytoma.

We identified two rare variants, c.553C>T (R185C) and c.1214T>A (L405Q), in *GALNT13* (Table 2). *GALNT13* variant c.553C>T (R185C) was detected in Family A in two affected siblings: one suffering from pilocytic astrocytoma (PA) (II-1) and the other from diffuse astrocytoma grade II (II-2) (Fig. 2). PA was diagnosed within the ages of 20–24 years, and astrocytoma grade II was diagnosed within the ages of 35–39 years. The variant was inherited from a father (I-1) with a family history of brain and other tumors. The genotyped unaffected sibling of the affected individuals (II-3) was a wild type for *GALNT13* c.553C>T (R185C). The other *GALNT13* variant, c.1214T>A (L405Q), was shared by two affected siblings (II-1 and II-2) in Family C (Fig. 2). Siblings were diagnosed with diffuse astrocytoma grade II (II-1) and PA (II-2) within the ages of 30–34 and 5–10 years, respectively. The PA-affected sibling (II-2) also had non-Hodgkin lymphoma. The variant was inherited from the father (I-1), who had a family history of brain and other tumors. Family history of other tumor types is presented for families carrying a *GALNT13* variant (combined tumor type frequencies of families Families A and C) in Supplementary Table 2. Both *GALNT13* variants were rare missense variants (frequency ≤ 0.00008 in the Finnish population) and were predicted to be damaging by multiple algorithms (8 out of 8) (Table 2). *GALNT13* c.553C>T (R185C) occurred within a functional protein domain on a substrate-binding site, and c.1214T>A (L405Q) was located on a conserved position (Table 2) outside this domain.

### *AR* variant detected in two families

*AR* c.2180G>T (R727L) was detected in two families in a total of three affected individuals (Table 2). The siblings having PA (II-1) and diffuse astrocytoma grade II (II-2) in Family A had the *AR* variant, which they inherited from the father (I-1), who was hemizygous for the variant (Fig. 2). The other parent had the wild type alleles (I-2). In addition, the unaffected female sibling (II-3) was a carrier. In Family Q, *AR* c.2180G>T (R727L) was observed as hemizygous in a man (II-1) whose diffuse astrocytoma grade II had been diagnosed within the ages of 20–24 years (Fig. 2). The variant was inherited from a mother (I-2) with a family history of brain and other tumors. The other parent (I-1) had the wild type for the variant. Family history of other tumor types is presented for families harboring the *AR* variant (combined tumor type frequencies of Families A and Q) in Supplementary Table 2. *AR* c.2180G>T (R727L) is a rare missense variant (frequency 0.008 in the Finnish population) located on a conserved region on a ligand binding domain, and it was predicted to have a damaging effect based on multiple algorithms (7 out of 7) (Table 2).

### *MYO10* variants detected in several families

*MYO10* c.4448A>G (N1483S) was observed in two families (Table 2). In Family B, the variant was shared by the affected parent (II-2) and the child (III-1) (Fig. 2). The parent had received an oligodendroglioma grade II diagnosis within the ages of 35–39 years, and the child was diagnosed with PA under the age of five years. The parent also had cervical cancer. The variant was inherited from the mother of the parent (I-1) with a family history of non-brain tumor types. In Family J, the GBM-affected family member (II-1) was identified to harbor the variant. GBM was diagnosed within the ages of 45–49 years. The unaffected father (I-1) had the wild type genotype, indicating that the variant was inherited from the family side with brain and other tumors. In both families, an unaffected sibling and/or a parent carried the variant. *MYO10* c.4448A>G (N1483S) is a rare missense variant (frequency 0.00009 in the Finnish population) on a PH2 domain and was predicted to be deleterious by algorithms (6 out of 7) (Table 2). It is also situated on a genomic *CTCF* binding site. We identified another rare germline variant on *MYO10* in Family F through WGS data (Table 2; Fig. 2). *MYO10* c.1511C>T (A504V) (frequency 0.002 in the Finnish population) was detected in a woman diagnosed with diffuse astrocytoma grade II within the ages of 30–34 years (II-1). Further genotyping of the sample cohort revealed that the unaffected father (I-1) had the wild type, indicating that the variant was inherited from the side of the family with brain and other tumors. Family history of other tumor types is presented for families carrying a *MYO10* variant (combined tumor type frequencies of Families B, J and F) in Supplementary Table 2. In addition, variant c.1511C>T (A504V) was detected in Family O in two unaffected relatives but not in a PA-affected family member. *MYO10* c.1511C>T (A504V) is a missense variant located on a conserved position on a myosin motor domain, and multiple computational algorithms predicted the variant to have damaging effects (7 out of 7) (Table 2). None of the discovered variants in *GALNT13*, *AR*, or *MYO10* were detected as germline variants in GBM, LGG, or PA patients of the PCAWG project. However, *MYO10* harbored four other potentially pathogenic rare germline variants (three missense, one stopgain, all detected in one individual) and *AR* one additional pathogenic variant (missense, detected in two individuals) in the PCAWG cohort (Supplementary Table 3). The variants detected in our Finnish glioma family cohort are more frequent in the Finnish population compared to non-Finnish Europeans and global population (Table 2).

Interestingly, in addition to *MYO10* c.1511C>T (A504V), II-1 in Family F carried a somatic tumor mutation *MYO10* c.745C>T (R249X) (Supplementary Table 4). The mutation creates a stop codon and is located on the myosin motor domain coding region. In addition to *MYO10* c.745C>T (R249X), five other somatic *MYO10* frameshift or missense mutations were detected in the Finnish familial glioma tumor cohort (Supplementary Tables 1 and 4). A diffuse astrocytoma grade II patient from Family L carried two somatic missense mutations, c.4962A>C (K1654N) and c.4628C>T (P1543L), and a frameshift mutation, c.1571_1587del (L524Qfs*1). *MYO10* c.4962A>C (K1654N) is located on the MyTH4 domain, and c.1571_1587del (L524Qfs*1) is located on the myosin motor domain. Somatic frameshift mutation *MYO10* c.4554_4638del (N1519Ffs*5) and missense mutation c.4682G>T (G1561V) were identified in a GBM tumor in Family H. The frameshift c.4554_4638del (N1519Ffs*5) is located between a PH2 and a C-terminal MyTH4/FERM domain, and c.4682G>T (G1561V) is situated on the MyTH4 domain. Furthermore, *GALNT13*, *AR*, and *MYO10* were somatically altered at low frequency in public diffuse glioma cohorts^17,18^. In the TCGA diffuse glioma cohort, one GBM tumor harbored homozygous deletion of *GALNT13* and six IDH-mutant astrocytomas amplification in *AR*^15,17,18^. In the GLASS consortium data, a frameshift mutation was present in *GALNT13* (A223Qfs*3, in a GBM) and *MYO10* (G672Afs*17, in an IDH-mutant astrocytoma)^16–18^. In addition, missense and other point mutations were reported in both cohorts; they differed from the ones detected in our analyses.

### Variants in *TP53*

The majority of the *TP53* variants (11 out of 12) were identified in at least one affected glioma patient (Supplementary Table 5). The detected SNPs were intronic, synonymous or located on the 3´UTR. In addition, an intronic 16-nt indel was identified. All identified variants are reported in the NCBI dbSNP ^19^.

Rare *TP53* variants—c.993+12T>C, c.376-86T>C, and c.108G>A (Pro36 =) (Finnish population frequency 0.003–0.004), which are in linkage disequilibrium (LD) with each other (D´ = 1.0, r^2^ = 1)—were detected in the affected individual in Family O (Supplementary Table 5, but the variants were not inherited from the family with history of brain tumors. *TP53* c.108G>A (Pro36 =) is a synonymous SNP, while c.376-86T>C and c.993+12T>C are intronic variants.

*TP53* glioma GWAS variants c.*1175A>C^8,20^, which has been identified to account for ∼6% of the familial risk of glioma^20^, and its lead SNP c.376-117G>A^21^ (variant frequency 0.02 and 0.03 in the Finnish population, respectively), were identified in Family P in the two affected siblings having oligodendroglioma grade II and ganglioglioma grade I within the ages of 10–14 and 30–34 years, respectively (Supplementary Table 5). The oligodendroglioma-affected sibling had also been diagnosed with meningioma, melanoma and colon cancer, and ganglioglioma-affected sibling with basal cell carcinoma of the skin. Two of the unaffected siblings in Family P carried both or one of the variants. In addition, an unaffected family member in Family N was a carrier for these SNPs, but the glioma-affected individual in the same family had the wild type. Furthermore, a total of five common *TP53* variants (Finnish population frequency > 0.05) were identified in the study cohort (Supplementary Table 5).

Based on the available pathogenicity annotations (CADD and/or ClinVar) and conservation scores for intronic, 3´UTR and synonymous variants, the *TP53* variants, except *TP53* c.*1175A>C, were predicted to be benign (Supplementary Table 5). The GWAS variant *TP53* c.*1175A>C, which is located on the *TP53* polyadenylation signal, was associated with reduced *TP53* expression and apoptosis levels by Li et al.^22^.

### Variants identified by a complementary WES variant analysis

We detected one or two candidate variants in each exome-sequenced family (A, B, C, D) with the filtering approach that prioritized very rare variants, which were predicted to be damaging, in genes relevant in glioma and brain based on the published literature (Supplementary Table 6). Variants of *STAT4*, *IGF1R*, *ENO2*, *NOS2* and *ASPM* were also identified by the above WES data-filtering approach and therefore genotyped using targeted sequencing in the additional 15 families with brain tumors. All variants were unique to the exome-sequenced families.

### Previously reported glioma-risk variant in *CCDC26* and other common glioma-associated variants detected in several families

In addition to the variants detected in WES analysis (Supplementary Table 7), we analyzed with targeted sequencing 102 variants which have been previously associated with glioma risk, called hereafter as literature variants. Approximately 56% (57 out of 102) of these literature variants were detected in at least one familial glioma patient, and two variants were detected only in unaffected family members. These variants were mostly intronic (n = 26), missense (n = 15) or downstream gene (n = 7) variants (Supplementary Table 8).

The known pathogenic frameshift variant *CHEK2* c.1100delC (rs555607708, chr22:29091856) (frequency 0.009 in the Finnish population) was detected in two families (Supplementary Table 8). In Family I, a glioma-affected individual and an unaffected sibling carried the variant. The glioma patient had been diagnosed with oligodendroglioma grade II within the ages of 30–34 years. The variant was not inherited from the brain tumor side of the family. In addition, *CHEK2* c.1100delC was identified in Family R, in an unaffected spouse of an unaffected individual, who was not carrying c.1100delC and belonged to the family with brain tumors. No samples were available from the affected individuals of this family.

The targeted sequencing included the 15 glioma GWAS variants reported in Melin et al.^8^ and/or Kinnersley et al.^23^ (Supplementary Table 8). These GWAS variants were common in the Finnish population (population frequency 0.10–0.80, median 0.41). Variants were identified in two to 16 of the studied families. Among the detected variants was the *CCDC26* intronic SNP (rs55705857, chr8:130645692 in the hg19 and chr8:129633446 in the hg38 genome)) (Finnish population frequency 0.09), which has been associated with an approximately sixfold relative risk of developing oligodendroglioma or IDH-mutant astrocytoma^24,25^. The variant frequency in the Finnish population is higher (0.09) compared to other populations (frequency in all populations combined: 0.04, frequencies in populations other than Finnish population: 0.0008–0.06). The SNP was detected in eight affected individuals belonging to six different families (I, B, Q, C, E, L) of which three families (B, Q, C) also carried rare *GALNT13*, *MYO10* or *AR* variants (Fig. 2). In all these three families, *CCDC26* rs55705857 SNP co-occurs with a variant in *GALNT13*, *MYO10* or *AR* (Fig. 2). A total of seven affected individuals carried the variant as heterozygous, and one affected case (Family Q) was homozygous for the SNP (Fig. 2). A heterozygous genotype was detected in one individual having *IDH* mutant diffuse astrocytoma (grade II) (Family C) (Fig. 2) and in two individuals having oligodendroglioma (grade II) [Families B (Fig. 2) and I]. Other affected individuals had been diagnosed with pilocytic astrocytomas (n = 3) or astrocytoma grade II tumors (IDH wild type based on tumor WGS) (n = 2). The variant was detected only in unaffected family member(s) in six additional families. In addition to the *CCDC26* variant, analyzed families with brain tumors harbored GWAS variants, e.g. in the genes *TERT*, *EGFR*, *CDKN2B-AS1*, *PHLDB1*, and *RTEL1* [variant frequency 0.23–0.80 (median 0.57) in the Finnish population] (Supplementary Table 8), which have been reported to confer a slightly increased risk of glioma^26^.

Altogether 14 common GWAS variants [variant frequency 0.10–0.80 (median 0.43) in the Finnish population] were detected in the families (variant detected in at least one affected genotyped family member), which carried rare, predicted damaging variants of *GALNT13*, *AR* or *MYO10* (Family A n = 9 GWAS variants, Family B n = 13, Family C n = 10, Family F n = 11, Family J n = 8, Family Q n = 13). Except for *CCDC26* SNP [rs55705857; odds ratio (OR) 6.3 for oligodendroglioma and OR 3.4 for non-GBM tumors^23^], these GWAS variants are low-risk glioma variants (OR 1.2–1.5^23^). In addition to the six families, other 11 Finnish families with brain tumors carried the 15 GWAS variants, which were targeted in this study, (n = 7–12 variants per family) (variant detected in at least one affected genotyped family member). Polygenic risk scores of GWAS variants did not differentiate affected individuals from unaffected ones, also when families with *CCDC26* SNP rs55705857 were excluded from the analysis (Supplementary Information).

A chromosomal region on 17q has been linked with familial glioma^27^. We successfully genotyped a total of 19 of the 21 candidate variants at 17q^28^ in Finnish glioma families, but none of the variants explained the familial aggregation of cases. Similarly, no familial glioma-linked variants of *POT1*^29^ were detected. One out of the three originally described variants of *POT1* (HG19:chr7:g.124481048C>A)^29^ was unsuccessfully genotyped in targeted sequencing, but based on WES, none of the four families carried the variant.

We analyzed somatic loss of heterozygosity events in eight glioma tumor samples from individuals belonging to eight families (D, F, G, H, C, L, B, Q) (Supplementary Table 1) on variant-carrying genomic regions selected based on the WES data as well as on *TP53* and literature variant regions, but no significant loss of heterozygosity was detected with regard to the identified variants in any of the patients.

## Discussion

While most brain tumors are sporadic, there is an increased risk for developing brain tumors among relatives of patients, suggesting inherited susceptibility^5^. The Finnish origin of the studied families facilitates the identification of genetic risk factors^30^. The relatively homogenous Finnish population is an interesting cohort for studying brain tumor risk factors and familial aggregation also because the prevalence of CNS tumors is particularly high in Finland and other Nordic countries^31^, which has not been explained by any environmental risk factors. Our Finnish familial glioma cohort suits well for discovering hereditary factors underlying the risk of glioma, because most gliomas in the cohort are LGGs, which have been observed to be associated with increased familial risks in registry studies by us and others^7,32,33^. Furthermore, based on the Finnish population-based registry study, the risk is increased for early-onset CNS tumors in first-degree relatives of early-onset CNS tumor patients^34^. CNS tumor subtype analysis revealed that the risk is increased, particularly for the relatives of early-onset nonsyndromic diffuse grade II–III glioma patients^7^. Here, we conducted a family-based study to identify novel and previously reported variants that increase the risk for nonsyndromic familial gliomas. Using an exome-based approach, we identified candidate gene variants, which were further analyzed in a total of 19 Finnish families with brain tumors. The study identified rare variants with predicted functional significance in *GALNT13*, *AR* and *MYO10*. The unique genetic nature of the Finnish population might explain why these variants have not been reported in other populations.

We observed two rare amino acid-changing variants in *GALNT13*, c.553C>T (R185C) and c.1214T>A (L405Q), in families A and C, respectively. The variants were unique to the families but shared between affected cases within families. Unaffected parents of index cases of the families were variant carriers, indicating incomplete penetrance of the variant. Incomplete penetrance refers to the situation in which some individuals who carry the pathogenic variant express the associated trait while others do not^35^. *GALNT13* is a member of a GalNAcT protein family and functions in mucin-type O-glycosylation of peptides. *GALNT13* c.553C>T (R185C) is located in a glycosyltransferase region on a catalytic subdomain A at a substrate-binding site and is predicted to be damaging. *GALNT13* c.1214T>A (L405Q) is located at a conserved genomic position outside functional domains of the protein and is predicted to be pathogenic, but the exact effect of the variant on protein function remains unclear. *GALNT13* is exclusively expressed in the brain and overexpressed in fetal brain^36^ as well as nestin-positive neural progenitor cells^37^, which are overpopulated in IDH-mutant LGGs^38,39^. *GALNT13* contributes to neural and neuronal cell differentiation, but is not expressed in mature astrocytes^36,37^. In gliomas, *GALNT13* is overexpressed in LGGs based on database analysis^40^. Similarly, Ducray et al. found *GALNT13* to be overexpressed in 1p19q codeleted oligodendrogliomas compared to normal brain or *EGFR* amplified cases representing IDH wild type GBMs^41^.

A total of two families (A and Q) carried the rare *AR* c.2180G>T (R727L) variant. The variant was shared between the affected cases in Family A, but unaffected family members also carried the variant in both families, demonstrating incomplete penetrance for the allele. *AR* functions as a steroid hormone-activated transcription factor and is the main driver of prostate cancer^42^. *AR* c.2180G>T (R727L) is located on a transactivation domain within a ligand binding domain on the *AR* gene and inhibits SIAH2 binding. AR-SIAH2 binding has been reported to modulate AR-mediated transcriptional regulation through degradation of selected AR-protein complexes^43^. *AR* binding decreases the E3 ubiquitin ligase activity of SIAH2^44^, so the activity of SIAH2 might also be increased due *to AR* c.2180G>T (R727L). *SIAH2* encodes a RING-type ubiquitin E3 ligase, which has been shown to have both oncogenic and tumor-suppressive roles in tumorigenesis and metastasis in several cancers^45^. *AR* has been observed to promote GBM tumorigenesis via somatic gene amplification and overexpression^46^, and it was recurrently amplified in IDH-mutant astrocytomas in TCGA data. Interestingly, *AR* c.2180G>T (R727L) was detected in unrelated and familial Finnish prostate cancer patients^47^. All affected individuals belonging to two prostate cancer families shared the variant, while unaffected family members had the wild type for the variant. The authors concluded that *AR* c.2180G>T (R727L) could confer an up to sixfold increased risk of prostate cancer and account for cancer development in up to 2% of Finnish prostate cancer patients. Based on CAG repeat analysis of prostate cancer patients, the *AR* c.2180G>T (R727L) variant originates from a single ancestral event^47^. Based on germline WGS data, *AR* c.2180G>T (R727L) variant-positive diffuse astrocytoma affected individual II-2 in Family A had 22 and 25–26 CAG repeats, consistent with the findings of Mononen et al.^47^.

Our study detected two rare germline variants, c.4448A>G (N1483S) and c.1511C>T (A504V), of *MYO10* in the familial glioma cohort. *MYO10* c.4448A>G (N1483S) was shared by the affected parent and offspring in Family B, and it was detected in the affected individual in Family J. *MYO10* c.1511C>T (A504V) was identified in Family F in the index case. Unaffected family members also carried either one of these variants, suggesting incomplete penetrance. *MYO10* is a member of a superfamily of motor proteins and is best known for its function in filopodia formation^48^, axon outgrowth and branching^49^. The predicted pathogenic *MYO10* c.4448A>G (N1483S) is located on the PH2 domain, which mediates PIP3 binding^50^. Disruption of the interaction between the domain and PIP3 inhibits filopodial formation and elongation^50^. Similarly, *MYO10* c.1511C>T (A504V) is predicted to be pathogenic and is situated on the myosin motor domain, which is essential for the formation of filopodia^51^. Interestingly, somatic *MYO10* mutations were observed in tumors of familial glioma patients, further supporting the role of *MYO10* in tumor development. *MYO10* is overexpressed in *EGFR*-overexpressing GBMs^52^, and GBM mouse models indicate that *MYO10* supports malignancy because knockout of *MYO10* impairs GBM invasion, slows tumor proliferation, reduces integrin-related signaling and prolongs survival^53^. High *MYO10* expression has been associated with poor prognosis and correlates with metastatic capacity in other tumor types^54,55^. *MYO19*, which belongs to the myosin protein family, has been reported as a candidate gene for familial glioma^28^.

No known cancer syndrome gene was identified to explain the familial aggregation of gliomas in the families. All coding regions and exon–intron boundaries of *TP53* were sequenced to exclude the possibility of Li-Fraumeni syndromic gliomas. No rare (variant frequency < 0.01 in the Finnish population) pathogenic variants of *TP53* were detected, consistent with our previous study, in which specific exons of *TP53* were screened from the family cohort^12^. The familial breast and ovarian cancer high risk variant *CHEK2* 1100delC, which is also connected to Li-Fraumeni syndrome^56^, was detected in one affected individual in Family I, but it did not explain the aggregation of glioma cases, because the variant was not inherited from the parent with family history of brain tumors.

Family-based WES is a powerful tool to detect rare, high-risk coding variants that disrupt gene function, although variants outside exonic regions are largely not covered and have been missed also in this study. However, the inclusion of previously reported variants, the majority of which are non-exonic, in the targeted sequencing analysis partly compensates for this limitation. To pinpoint germline alterations which are likely to be associated with increased risk of familial glioma, instead of a statistical approach, we applied comprehensive variant filtering strategy and computational annotation including variant segregation in Finnish glioma families, use of population frequencies, multiple functional impact scores and variant pathogenicity predictions as well as information of gene relevance to glioma. Scientific knowledge and analysis methods are constantly updating, and the results and conclusions of this study are based on the current knowledge.

We wanted to have a blood sample from at least two affected individuals in the WES families and samples from additional cases for estimating the segregation. This gave us good grounds for filtering and analyzing the variants. However, selecting genomic regions for targeted sequencing based on the WES results likely means that we are not able to detect all the relevant variants in Finnish glioma families, as also indicated by the power calculation. As an example, several private variants, like those in genes STAT4, IGF1R, ENO2, NOS2, ASPM and NPC1, were detected. These variants may contribute to the glioma risk in these specific families. Detailed analysis of private variants could direct the search for correct pathways disrupted in familial gliomas, however, it was not within the scope of the study. Furthermore, we cannot fully presume that all the families with more than one glioma case carry risk variants in their germline, although gliomas are very rare and this is extremely unlikely by chance. We prioritized variants and variant-carrying genes detected in at least two families to increase the confidence of their relevance. In any case, functional studies are needed to validate the causality of the alternative variants and candidate genes and to characterize the cellular mechanisms contributing to increased glioma risk. However, it is challenging to experimentally evaluate the functional effect of the discovered rare variants on glioma development, as the penetrance of the detected variants was not complete and their functional impact is highly context dependent. Thus, it is challenging and laborious to generate suitable research models for these studies.

The known glioma risk variant rs55705857 in *CCDC26* was co-inherited together with rare variants in *GALNT13*, *MYO10* or *AR* in three out of six families carrying these rare variants, and it was also detected in affected cases in three additional families. In previous studies, rs55705857 has been associated with an approximately sixfold relative risk of developing IDH-mutant astrocytoma or oligodendroglioma^24,25^. Furthermore, it has been linked to early disease onset^57^, which is reflecting our findings that early onset non-GBM diffuse gliomas are associated with increased risk among patient’s relatives^7^. In addition to tumor types that likely represent these IDH-mutant LGGs, rs55705857 variant was also detected in three pilocytic astrocytoma patients in our analysis, suggesting that its association with pilocytic astrocytoma could be investigated also in other cohorts. The functional effect of this variant has been elucidated by previous studies. It has been shown that the variant increases *Myc* expression by disrupting OCT2/4 binding to the DNA due to base substitution (A>G), which allows increased interaction with *Myc* promoter and *Myc* overexpression^25,58^. rs55705857 resides in an enhancer region which is active during neural and brain development (especially in radial glial stem cells and a subset of oligodendrocyte precursor cells) and variant increases the activity of this regulatory region^25^. Interestingly, also *GALNT13* and *AR* have been linked to neural cell development and the regulation of neural progenitor cells in previous studies^36,37,59,60^.

When considering the rate, inheritance pattern, and population frequencies of detected rare, damaging coding variants in *MYO10*, *AR* and *GALNT13*, enhancer-linked risk variant in *CCDC26*, and other detected risk variants, our results suggest polygenic inheritance of familial glioma in Finland. rs55705857 allele frequency is 9% in the Finnish population, and this variant cannot fully explain the increased risk in our familial glioma cohort. The reported allele frequency of rs55705857 is higher in Finland than among non-Finnish Europeans and in the global population, but this is also true for the discovered rare variants in *MYO10*, *AR* and *GALNT13*. The evaluation of the detected variants as clinical biomarkers as part of family-based risk management is warranted.

### Supplementary Information

Supplementary Information.

Supplementary Tables.

## Data Availability

The datasets generated and analyzed during the current study are not publicly available due to lack of patient consent to deposit the data in public repositories. The data is available from the corresponding authors on reasonable request and with permission from data owners. The corresponding authors can be contacted for data requests. The sequencing coverage and quality statistics of WES, targeted sequencing, and WGS data are presented in Supplementary Table 9. The PCAWG project data used in this study can be retrieved from the ICGC data portal ^13,14^.
