# Supplementary material for: Previously reported CCDC26 risk variant and novel germline variants in GALNT13, AR, and MYO10 associated with familial glioma in Finland
Source: Sci Rep. 2024 May 21;14:11562. doi: 10.1038/s41598-024-62296-5 (PMC11109329; doi:10.1038/s41598-024-62296-5)
Supplement: Supplementary file 1 — Supplementary Information. [file 41598_2024_62296_MOESM1_ESM.docx]

Previously reported *CCDC26* risk variant and novel germline variants in *GALNT13*, *AR*, and *MYO10* associated with familial glioma in Finland

Riikka Nurminen^1^*, Ebrahim Afyounian^1^*, Niina Paunu^2^, Riku Katainen^3^, Mari Isomäki^1^, Anssi Nurminen^1^, Mauro Scaravilli^1^, Jenni Tolppanen^1^, Vidal Fey^1^, Anni Kivinen^1^, Pauli Helén^4^, Niko Välimäki^3^, Juha Kesseli^1^, Lauri A. Aaltonen^3^, Hannu Haapasalo^4,5^, Matti Nykter^1,6 +^, Kirsi J. Rautajoki^1,7+^

^1^ Prostate Cancer Research Center, Faculty of Medicine and Health Technology, Tampere University and Tays Cancer Center, Tampere University Hospital, Tampere, Finland

^2^ Department of Oncology, Tampere University Hospital, Tampere, Finland

^3^ Applied Tumor Genomics Research Program and Department of Medical and Clinical Genetics, University of Helsinki, Faculty of Medicine, Helsinki, Finland

^4^ Faculty of Medicine and Health Technology, Tampere University and Tays Cancer Center, Tampere University Hospital, Tampere, Finland

^5^ Fimlab Laboratories ltd., Tampere University Hospital, Tampere, Finland

^6^ Foundation for the Finnish Cancer Institute, Tukholmankatu 8, Helsinki, Finland

^7^ Tampere Institute for Advanced Study, Tampere University, Tampere, Finland

* These authors contributed equally to this work.

+ Co-corresponding authors: Adj. Prof. Kirsi Rautajoki, PhD, BioMediTech unit, Faculty of Medicine and Health Technology, Tampere University, Arvo Ylpön katu 34, 33520 Tampere, Finland, [kirsi.rautajoki@tuni.fi](mailto:kirsi.granberg@tuni.fi) and Prof. Matti Nykter, D.Sc.(Tech), BioMediTech unit, Faculty of Medicine and Health Technology, Tampere University, Arvo Ylpön katu 34, 33520 Tampere, Finland, [matti.nykter@tuni.fi](mailto:matti.nykter@tuni.fi)

Matti Nykter<https://orcid.org/0000-0001-6956-2843>
Kirsi Rautajoki<https://orcid.org/0000-0001-6549-7810>

# Supplementary Figure 1


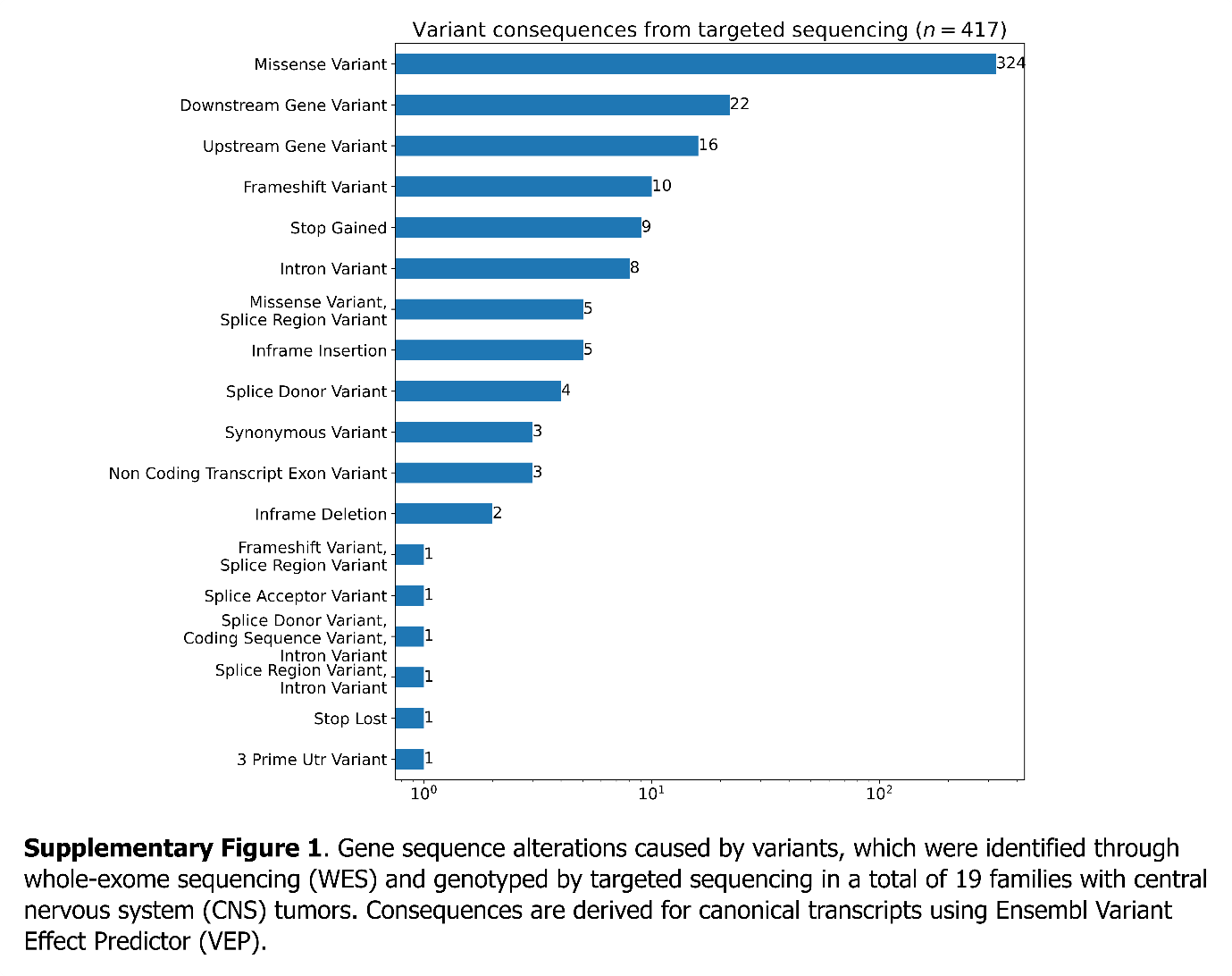


# Supplementary Methods

## *DNA sequencing and genotyping*

Whole exome sequencing (WES) was conducted by BGI Tech Solutions Co. Ltd. (Hong Kong, China). Libraries were prepared using an Agilent SureSelect V5 (50M) kit (Agilent, Santa Clara, CA, USA) and 90 bp paired-end sequenced with 50X genome coverage using Illumina’s HiSeq 2000 technology (Illumina, San Diego, CA, USA). A total of 76 variants identified through WES data were confirmed by Sanger sequencing. Briefly, target genomic regions were amplified using polymerase chain reaction (PCR) and sequenced with an ABI Prism Sequencer 3130xl instrument using the BigDye Terminator Cycle Sequencing Kit (Applied Biosystems, Foster City, CA, USA). Primer sequences and detailed PCR and sequencing conditions are available on request. Libraries were prepared for targeted sequencing using a TruSeq® Custom Amplicon Kit and sequenced on a MiSeq sequencer (Illumina) with ~220x coverage using 150 bp paired-end sequencing. WGS libraries were prepared using the Novogene NGS DNA Library Preparation Set and 150 bp paired-end sequenced with 30X (germline DNA) or 60X (FFPE DNA) coverage on the NovaSeq instrument (Illumina) at Novogene Co., Ltd. (Cambridge, UK). *MYO10* c.1511C>T was genotyped in 80 individuals belonging to the targeted sequencing cohort using TaqMan SNP Genotyping Assay (Applied Biosystems, Foster City, CA, USA), according to the manufacturer’s instructions with a QuantStudio 12K Flex Real-Time PCR system and software (Applied Biosystems).

## *WES* data analysis

Illumina base calling software, version 1.7, with default parameters was used for base calling, resulting in paired-end reads of length 90 bp. Adapter sequences in the raw data were removed, and low-quality reads having too many Ns and low base quality bases were discarded by BGI Tech Solutions. The resulting sequencing data were mapped to the human reference genome (hg19) using Bowtie2 software (version 2.2.5) [[1]](https://paperpile.com/c/FwxvfR/XYtH) and converted to BAM file format using Samtools (version 0.1.19) [[2]](https://paperpile.com/c/FwxvfR/F1Id). Duplicate reads were marked and removed from the resulting BAM files using SAMBLASTER (version 0.1.21) [[3]](https://paperpile.com/c/FwxvfR/D8hU). Two alternative tools, namely an in-house variant calling pipeline and the Genome Analysis Toolkit (GATK; Version=3.7-0-gcfedb67 [[4]](https://paperpile.com/c/FwxvfR/dQZa)), were used to separately detect germline variants, resulting in a list of raw variants.

Using the in-house variant calling pipeline, which was built upon *Samtools mpileup*, we set the parameters such that a site was called a *homozygous reference* if there were at least 8 supporting reads for the reference allele, and 95% of the reads showed the reference allele (i.e., --ref=8:0.95). A site was called *heterozygous* if there were at least 4 supporting reads for the alternate allele, and 25% of the reads showed the alternate allele (i.e., --hetz=4:0.25). A site was called *homozygous alternate* if there were at least 4 supporting reads for the alternate allele, and 80% of the reads showed the alternate allele (i.e., --homz=4:0.8). Controlling for technical errors due to, e.g., sequencing artifacts, in our in-house variant calling pipeline, we discarded the germline sites that were also found in a familial prostate cancer cohort consisting of a single family (n=9), as well as the germline sites from one patient with metastatic prostate cancer. We used Pearson's χ^2^ test to assess the probability that all samples had the same underlying allele fraction (indicating a sequencing error). We discarded the sites with a P-value > 0.0001. Additionally, we calculated a score for each site based on the following formula, and we discarded sites scoring less than 1:

$$site score = \prod_{i=1}^{m} ((n_{i}+1) * max(B(x_{i} ; n_{i}, 0.01), B(x_{i} ; n_{i}, 0.50), B(x_{i} ; n_{i}, 0.99)))$$

where *m* is the number of samples in the cohort; *n_i_* is the total number of reads at a site for the *i*th sample; *x_i_* is the number of reads with the alternate allele at a site for the *i*th sample; and *B* is the binomial discrete random variable probability mass function.

With regard to the GATK variant calling pipeline, we followed the Best Practices workflow retaining only those variants that achieved a FILTER status of PASS [[5]](https://paperpile.com/c/FwxvfR/Pe27). Variants with variant quality score log-odds (VQSLOD) smaller than 20 underwent manual inspection in Integrative Genomics Viewer (IGV).

Raw variants were annotated using ANNOVAR software (version 2014Jul14) [[6]](https://paperpile.com/c/FwxvfR/tL4C). Raw variants were filtered and selected as follows. Raw variants that were common (> 1% population allele frequency) in the Finnish population of the 1000 Genomes Project (n=100) [[7]](https://paperpile.com/c/FwxvfR/lqnW) or the Sequencing Initiative Suomi project (SISu, n=3325) [[8]](https://paperpile.com/c/FwxvfR/cg4q) were discarded. Based on the variants’ consequence annotations, variants with nonsynonymous, frameshift, stop-gain, and stop-loss consequences were retained. Other annotation information, such as pathogenicity scores, whether the gene harboring the variant was expressed in the brain tissue or glioma, and whether the variant was shared by the affected family members, was manually inspected to select prospective variants. Additionally, retained variants were inspected in the Integrative Genomics Viewer (IGV) [[9]](https://paperpile.com/c/FwxvfR/E8bB) to check for the presence of sequencing artifacts, and variants believed to be due to such artifacts were filtered out. Finally, variants meeting the filtration criteria in either set were combined. The final list of 417 WES variants that were further analyzed using the targeted sequencing data (see below) demonstrated high quality, as reflected by the QUAL scores reported by GATK, with the minimum QUAL value recorded at 291.05.

The results were manually inspected for *TP53* and *CHEK2* variants (typical in Li-Fraumeni syndrome). Additionally, the WES data from the samples underwent copy number alteration (CNA) analysis using CoNIFER software (version 0.2.2) [[10]](https://paperpile.com/c/FwxvfR/8bAg).

## *Targeted sequencing data analysis*

Variants in the targeted sequencing data were called using Illumina built-in data analysis software in the MiSeq instrument (i.e., MiSeq Reporter, TruSeq Amplicon workflow) with the default parameters. Bcftools software (version 1.9-151-gef6c0db) [[11]](https://paperpile.com/c/FwxvfR/klJu) was used to merge the gVCF files from samples, to extract the genotype calls for the loci of interest, and to convert the results into table format.

A total of 113 variants were manually selected based on a literature search and included in the targeted sequencing experiment. Peer-reviewed journal articles from 2010 until 2017 containing the word "glioma" were searched. These articles included, e.g., GWASs and GWAS meta-analyses. The resulting articles were shortlisted to those deemed relevant to the current study. The genomic positions of variants reported in these studies were extracted to be genotyped in our cohort. In addition, entries related to glioma were searched on the Online Mendelian Inheritance in Man (OMIM) (<https://www.omim.org/>), as well as SNPedia (<https://www.snpedia.com/index.php/SNPedia>), and the relevant genomic coordinates were extracted to be genotyped in our cohort. Furthermore, a few known cancer syndrome variants were included. In addition to genomic regions covering variants in our WES discovery cohort and the listed literature, all exons and exon-intron boundaries of *TP53* were included in targeted sequencing.

A total of 627 variants were called from targeted sequencing data. Variants having a call rate ≤ 0.9 (total read count < 5) across all individuals were excluded for quality control (QC). In addition, *TP53* variants were filtered out based on a low read count supporting the variant (n < 3) or allele fraction (< 0.15). A total of 12 variants were removed based on discordant WES and targeted sequencing calls. All of the samples had a call rate > 99% for the remaining variants (n=531). Variants were prioritized based on the following information: number of families in which variants were detected, whether variants were shared between affected family members and/or inherited from the brain tumor side of the family, Finnish population allele frequency, gene information, predicted pathogenicity (SIFT, PolyPhen, LRT, MutationTaster, MutationAssessor, FATHMM_MKL, MetaSVM, MetaLR), conservation (GERP++, phyloP, SiPhy), ClinVar [[12]](https://paperpile.com/c/FwxvfR/DoAb) pathogenicity annotation (review status ≥ 2/4 stars, i.e., criteria provided, multiple submitters, no conflicts), variant consequences and the association of a variant with gene expression. Ensembl Variant Effect Predictor (VEP) [[13]](https://paperpile.com/c/FwxvfR/vdOO) and ANNOVAR [[6]](https://paperpile.com/c/FwxvfR/tL4C) were used for annotation. Variant localization with regard to protein domains and ligand binding sites was derived from UniProt (<https://www.uniprot.org/>) [[14]](https://paperpile.com/c/FwxvfR/Quec). Variant associations with gene expression and gene expression in brain sections were derived from the GTEx Portal (<http://www.gtexportal.org/>) [[15]](https://paperpile.com/c/FwxvfR/hlsC). A gene was considered as expressed when TPM value was ≥ 1.0 in at least one of the 13 brain sections. A total of 36.3% of the 56200 transcripts available in GTEx Portal were determined to be expressed in the brain according to this criterion. Threshold of TPM value 1.0 was used, because some of the genes with stable protein expression, like TFs, can have low RNA expression values. In addition, a gene might be relevant although it is expressed only in part of the cells. Gene expression in glioblastoma (GBM) tumors and low-grade gliomas (LGGs) was derived from OncoDB (<http://oncodb.org/>) [[16]](https://paperpile.com/c/FwxvfR/iCf6). The OncoDB Differential Expression tool was used to define differential expression using default settings, i.e., FDR Adjusted P-value < 0.001 and |log2FC| > 1, and a difference between median expression > 50 units. Linkage disequilibrium (LD) of TP53 variants was determined using LDlink (CEU and FIN populations) (<https://ldlink.nci.nih.gov/>) [[17]](https://paperpile.com/c/FwxvfR/B5lV).

## *Complementary WES data analysis*

The selection of candidate variants was performed with BasePlayer software [[18]](https://paperpile.com/c/FwxvfR/oCE0). We included all very rare variants (allele frequency < 0.001 in the Finnish and all populations of gnomAD r2.1 exomes), which were shared by cases and were not present in unaffected individuals from the same family. In a family with two unaffected siblings genotyped (Family D), we also included variants that were present in only one of the samples from the unaffected siblings. We included all synonymous and nonsynonymous variants in all genes (Ensembl genes v87). Prioritization and effect prediction of variants were performed with VarElect [[19]](https://paperpile.com/c/FwxvfR/URAl) and VarSome [[20]](https://paperpile.com/c/FwxvfR/IBe1), respectively.

## *WGS data analysis*

Sequence reads were aligned to the human genome reference hg38. Aligned paired-end read variant calling followed GATK best-practices guidelines using GATK software, version 4.1.8.1 [[21]](https://paperpile.com/c/FwxvfR/27ey). Germline SNPs were analyzed using GATK/Haplotypecaller, and somatic mutations were analyzed using GATK/Mutect. Discovered alterations were annotated with ANNOVAR. Somatic loss of heterozygosity (LOH) regions were analyzed using Battenberg-hg38 [[22]](https://paperpile.com/c/FwxvfR/hccW). For comparisons with hg19 results, Picard tools LiftoverVCF (version 2.26.11) was used for lifting over genomic coordinates from the hg38 reference genome to hg19 (<https://broadinstitute.github.io/picard/>).

## *Power calculation for the WES data analysis*

We performed a power calculation for detecting a variant shared by both affected participants within each family, under the condition that the variant is found in at least one family and a specified fraction of familial-glioma families have the variant. We based the calculation on selecting four out of 19 families in our cohort for WES. To achieve this, we employed the following formula:

$power =1 - P(X=0) = 1- f(0; N, K, n)$,

where $P(X=k) = f(k; N, K, n)$ denotes the hypergeometric probability mass function, with N=19 (the total number of families), n=4 (the number of families selected for WES), K=N*ν (the number of families expected to carry the variant based on the fraction of familial-glioma families that have the variant) where ν is fraction of familial-glioma families that have the variant, and k >= 1 (the minimum number of families in which the variant must be found for detection). The WES data in this study has an average depth of coverage of 50X. The detection probability at 50X coverage, and taking into account the reference bias, is >99.98% [23]. This is supported by the observation that the validation rate for NGS variant calls is extremely high—99.965% [24]. Therefore, we did not incorporate the detection probability based on sequencing depth in the formula above as it was close to 1 for our experiment. Supplementary Figure 2 represents the result of power analysis across different variant frequencies.


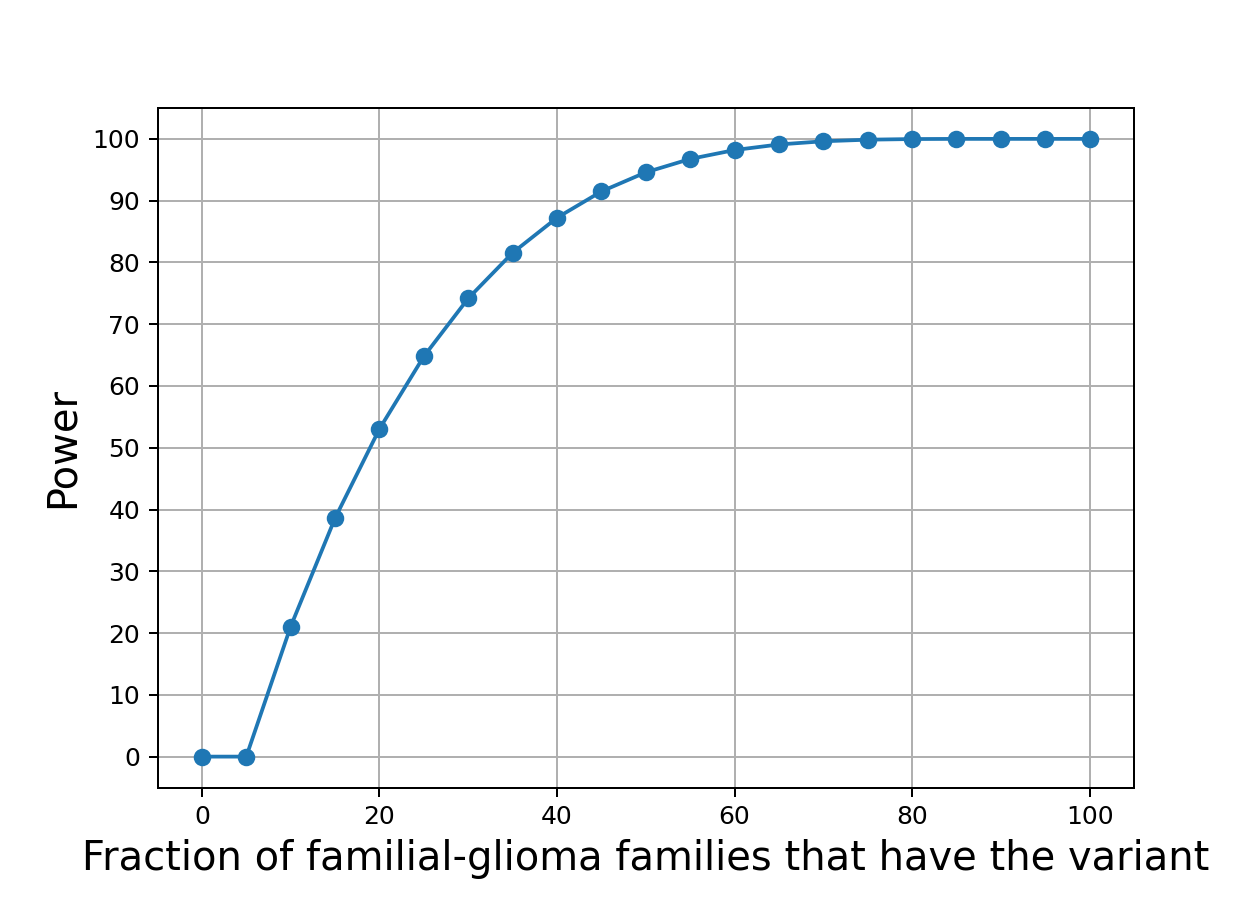


**Supplementary Figure 2**. Detection power at different fractions of familial-glioma families that have the variant.

## *Polygenic risk score analysis*

To assess the cumulative effect of GWAS variants included in our study, we computed polygenic risk scores (PRSs) for both the entire targeted sequencing cohort and a sub-cohort that excludes families carrying the rs55705857 variant. For this purpose, we gathered odds-ratios and p-values for the GWAS variants from their respective publications [25-28]. We omitted rs1412829 due to its linkage disequilibrium with rs4977756 (D´=0.8148,r^2^ = 0.645) in the Finnish population and excluded rs498872 because its p-value was 0.23 for increased risk for glioma. The odds ratios were transformed using the common logarithm to effectively account for both risk and protective alleles. Subsequently, we applied the Mann–Whitney U test to evaluate if there were distinguishable PRS scores between affected and unaffected participants. Our analysis revealed no statistically significant differences in PRSs between these two groups (p-values of 0.8985 and 0.8478 for whole cohort and when excluding families with rs55705857, respectively), indicating that the cumulative effect of the GWAS variants included in our study cannot differentiate the affected cases from unaffected ones in these families. The outcomes of the PRS calculations are illustrated in Supplementary Figure 3.


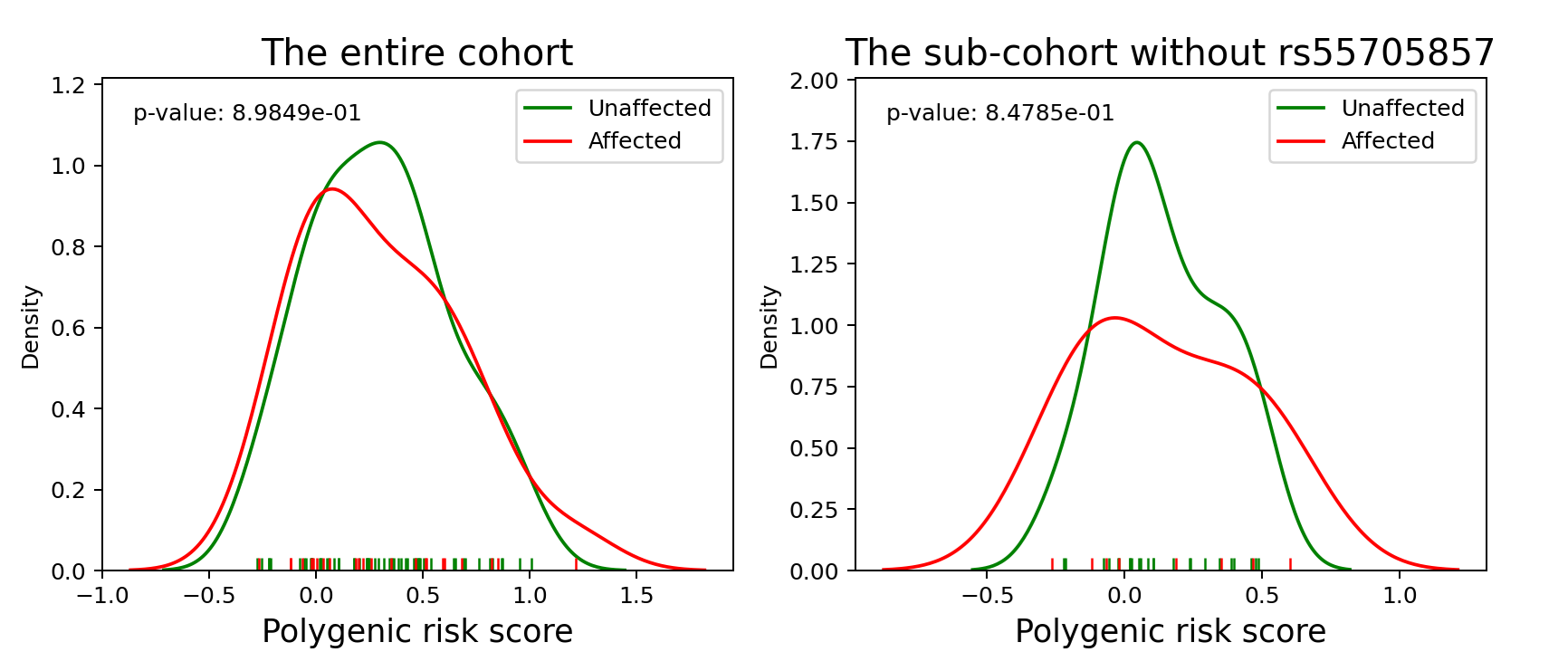


**Supplementary Figure 3**. Calculated polygenic risk scores suggest that the cumulative effect of the GWAS variants included in our study cannot differentiate the affected cases from unaffected ones within the studies families. The ticks show the individual polygenic risk scores per participant.

# References

1. Langmead B, Salzberg SL. Fast gapped-read alignment with Bowtie 2. Nat Methods. 2012;9:357–9.

2. Li H, Handsaker B, Wysoker A, Fennell T, Ruan J, Homer N, et al. The Sequence Alignment/Map format and SAMtools. Bioinformatics. 2009;25:2078–9.

3. Faust GG, Hall IM. SAMBLASTER: fast duplicate marking and structural variant read extraction. Bioinformatics. 2014;30:2503–5.

4. McKenna A, Hanna M, Banks E, Sivachenko A, Cibulskis K, Kernytsky A, et al. The Genome Analysis Toolkit: a MapReduce framework for analyzing next-generation DNA sequencing data. Genome Res. 2010;20:1297–303.

5. Van der Auwera GA, Carneiro MO, Hartl C, Poplin R, Del Angel G, Levy-Moonshine A, et al. From FastQ data to high confidence variant calls: the Genome Analysis Toolkit best practices pipeline. Curr Protoc Bioinformatics. 2013;43:11.10.1–11.10.33.

6. Wang K, Li M, Hakonarson H. ANNOVAR: functional annotation of genetic variants from high-throughput sequencing data. Nucleic Acids Res. 2010;38:e164.

7. 1000 Genomes Project Consortium, Abecasis GR, Auton A, Brooks LD, DePristo MA, Durbin RM, et al. An integrated map of genetic variation from 1,092 human genomes. Nature. 2012;491:56–65.

8. Sequencing Initiative Suomi project (SISu), Institute for Molecular Medicine Finland (FIMM), University of Helsinki, Finland. SISu. <http://sisuproject.fi.> [Accessed 2014.](http://paperpile.com/b/FwxvfR/cg4q)

9. Robinson JT, Thorvaldsdóttir H, Winckler W, Guttman M, Lander ES, Getz G, et al. Integrative genomics viewer. Nat Biotechnol. 2011;29:24–6.

10. Krumm N, Sudmant PH, Ko A, O’Roak BJ, Malig M, Coe BP, et al. Copy number variation detection and genotyping from exome sequence data. Genome Res. 2012;22:1525–32.

11. Danecek P, Bonfield JK, Liddle J, Marshall J, Ohan V, Pollard MO, et al. Twelve years of SAMtools and BCFtools. Gigascience. 2021;10.

12. Landrum MJ, Lee JM, Benson M, Brown GR, Chao C, Chitipiralla S, et al. ClinVar: improving access to variant interpretations and supporting evidence. Nucleic Acids Res. 2018;46:D1062–7.

13. McLaren W, Gil L, Hunt SE, Riat HS, Ritchie GRS, Thormann A, et al. The Ensembl Variant Effect Predictor. Genome Biol. 2016;17:122.

14. UniProt Consortium. UniProt: the universal protein knowledgebase in 2021. Nucleic Acids Res. 2021;49:D480–9.

15. GTEx Consortium. Human genomics. The Genotype-Tissue Expression (GTEx) pilot analysis: multitissue gene regulation in humans. Science. 2015;348:648–60.

16. Tang G, Cho M, Wang X. OncoDB: an interactive online database for analysis of gene expression and viral infection in cancer. Nucleic Acids Res. 2022;50:D1334–9.

17. Machiela MJ, Chanock SJ. LDlink: a web-based application for exploring population-specific haplotype structure and linking correlated alleles of possible functional variants. Bioinformatics. 2015;31:3555–7.

18. Katainen R, Donner I, Cajuso T, Kaasinen E, Palin K, Mäkinen V, et al. Discovery of potential causative mutations in human coding and noncoding genome with the interactive software BasePlayer. Nat Protoc. 2018;13:2580–600.

19. Stelzer G, Plaschkes I, Oz-Levi D, Alkelai A, Olender T, Zimmerman S, et al. VarElect: the phenotype-based variation prioritizer of the GeneCards Suite. BMC Genomics. 2016;17 Suppl 2:444.

20. Kopanos C, Tsiolkas V, Kouris A, Chapple CE, Albarca Aguilera M, Meyer R, et al. VarSome: the human genomic variant search engine. Bioinformatics. 2019;35:1978–80.

21. Van der Auwera GA, O’Connor BD. Genomics in the Cloud: Using Docker, GATK, and WDL in Terra. O’Reilly Media; 2020.

22. Nik-Zainal S, Van Loo P, Wedge DC, Alexandrov LB, Greenman CD, Lau KW, et al. The life history of 21 breast cancers. Cell. 2012;149:994–1007.

24. Li, C.-I., Samuels, D. C., Zhao, Y.-Y., Shyr, Y. & Guo, Y. Power and sample size calculations for high-throughput sequencing-based experiments. Brief. Bioinform. 19, 1247–1255 (2018).

25. Koboldt, D. C. Best practices for variant calling in clinical sequencing. Genome Med. 12, 91 (2020).

26. Shete, S. et al. Genome-wide association study identifies five susceptibility loci for glioma. Nat. Genet. 41, 899–904 (2009).

27. Jenkins, R. B. et al. A low-frequency variant at 8q24.21 is strongly associated with risk of oligodendroglial tumors and astrocytomas with IDH1 or IDH2 mutation. Nat. Genet. 44, 1122–1125 (2012).

28. Kinnersley, B. et al. Genome-wide association study identifies multiple susceptibility loci for glioma. Nat. Commun. 6, 1–9 (2015).

29. Rajaraman, P. et al. Genome-wide association study of glioma and meta-analysis. Hum. Genet. 131, 1877–1888 (2012).
